# Recruitment and Entrapment Pathways of Minors into Sex Trafficking in Canada and the United States: A Systematic Review

**DOI:** 10.1177/15248380211025241

**Published:** 2021-06-29

**Authors:** Kyla Baird, Jennifer Connolly

**Affiliations:** 1 Department of Psychology, 56014 York University, Toronto, Ontario, Canada

**Keywords:** domestic minor sex trafficking, commercial sexual exploitation of children, pathways, recruitment, entrapment, exploitation

## Abstract

The domestic sex trafficking of minors is occurring across Canada and the United States. Understanding the routes into sex trafficking, including the way traffickers target, recruit and enmesh youth in the sex trade is invaluable information for service providers and law makers developing prevention and intervention initiatives. This review synthesized research on the exploitation processes and tactics employed by traffickers in the sex trafficking of domestic minors in Canada and the US. The authors comprehensively and systematically searched five electronic databases and obtained additional publications and grey literature through a backward search of the references cited in articles reviewed for inclusion.  Inclusionary criteria included: Studies published in the English language between January 1990 and June 2020 containing original research with quantitative or qualitative data on the recruitment or pathways into sex trafficking for minors trafficked within the US and Canada. The search yielded 23 eligible studies. The synthesis of the studies in the review converged on the notion of sexual exploitation occurring on a continuum comprising of three components; the recruitment context, entrapment strategies utilized by traffickers, and enmeshment tactics used to prolong exploitation. Findings highlight the significant physical, psychological and emotional hurdles faced by youth victims of sex trafficking and point to the importance of comprehensive and holistic approaches to prevention and intervention practices.

Human trafficking is a global problem that has garnered significant international and national attention over the past 2 decades. In 2000, 140 countries signed onto the Palermo protocol agreeing that human trafficking is a significant human rights violation and a criminal offense that requires prevention, the protection of vulnerable populations, and the prosecution of violators of the protocol. In North America, both Canada and the United States signed this protocol and have since passed legislation and policies to combat human trafficking. Sex trafficking became criminalized in Canada in 2005 when human trafficking entered the criminal code under section 279.01 and in the United States in 2000 with the passing of the Trafficking Victims Protection Act (TVPA). Sex trafficking is one of the most common forms of human trafficking consisting of the recruitment and exploitation of an individual through the use of threats, force, coercion, deception, or abuse of power for the purpose of a commercial sex act (United Nations Office on Drugs and Crime, 2014). A commercial sex act, as defined by the American TVPA (2000), is “any sexual act for which something of value is given or received.” Common examples include prostitution, pornography, sexual massage parlors, and strip clubs. Commercial sex acts may be exchanged for money, drugs, shelter, clothing, or food (Cole & Anderson, 2013; Kotrla, 2010). Sex trafficking is rampant across the United States and Canada (Clawson et al., 2009; Dalley, 2010). Despite various political and social differences between these countries, they are united on the front of combating sex trafficking within their borders and expanding research to support effective evidence-based prevention and intervention strategies.

## Sex Trafficking of Minors (STM)

Minors (under the age of 18) are overrepresented among victims of sex trafficking, with the majority of victims recruited between 12 and 14 years of age (Jordan et al., 2013; Smith et al., 2009). Given the elevated risk for minors, research and legislation have begun to focus on the specific issue of the STM. Consequently, our understanding of the risks for recruitment, experiences, and needs of underage victims is growing, and important policy actions have been taken. In the past decade, both Canada and the United States have passed legislation, reformed laws, and enacted policies to combat issues of the STM. Legislative changes in both Canada and the United States have transformed the way victims are viewed and treated by law enforcement. More specifically, American and Canadian federal consent laws declared minors under the age of 18 unable to consent to commercial sex and have shifted the lens of law enforcement from criminalizing youth in the sex trade to viewing them as victims (Adelson, 2008; Franchino-Olsen, 2019). Language in research on STM has followed suit, shifting from calling underage victims of sex trafficking “teen prostitutes” to “victims of STM.”

On the basis of age, youth from all sectors of society are at risk for recruitment into sex trafficking. Developmental vulnerabilities such as identity formation, the need for belonging, desire for autonomy, desire for romantic relationships, and evolving problem-solving skills make them easily exploitable by traffickers who appeal to these vulnerabilities (Schwartz, 2015). Based on the growing literature, some youths are at greater risk for recruitment than others. Several risk factors for STM have been identified, including involvement with child protective services, history of childhood sexual abuse, homelessness, physical and emotional abuse, neglect, exposure to intimate partner violence, problematic relationships with caregivers, drug and alcohol abuse, and teen dating violence (Choi, 2015; Countryman-Roswurm, 2012; Countryman-Roswurm & Bolin, 2014; Farley et al., 2005; Franchino-Olsen, 2019; Kotrla, 2010; Landers et al., 2017). Traffickers are known to be deeply perceptive of the developmental vulnerabilities of youth and target their unmet needs through strategic recruitment methods.

Simply being a girl places a youth at an elevated risk status relative to boys (Estes & Weiner, 2001), with 98% of victims being women and girls (International Labour Organization, 2012). Adolescent girls are particularly vulnerable to sexual exploitation due to social norms that cast gendered expectations and power imbalances in relation to sexual activities, with boys being expected to take sexual initiatives. Sexual inexperience, desire for romantic relationships, and insecurity among young girls can set the stage for manipulation and exploitation by adolescent boys or men (Hanna, 2002).

Based on the differential needs and situations of youth, the recruitment and exploitation of underage populations are thought to differ from adult populations (Bouché & Shady, 2017; Dank et al., 2014). While it may be riskier to traffic a youth due to increased policing efforts in protecting minors and higher sentences for STM, it has been suggested that these risks are offset by the youth being easier to manipulate and control and being highly desired by purchasers, bringing in more money for the trafficker (Dank et al., 2014). Compared to adults, youths have greater needs for protection, less life experience, and are dependent on adults for basic needs such as food and shelter, making them more vulnerable to traffickers who vow to provide care, protection, and basic needs (Bruhns et al., 2018; Cole & Anderson, 2013). Given youths’ physical and emotional dependency on adults, some research have suggested youths are more trusting and less able to identify traffickers’ coercive and manipulative strategies to entrap them (Cole & Sprang, 2015). Adult victims, on the other hand, are generally less psychologically dependent on their trafficker (Bouché & Shady, 2017). In addition, literature on the trafficking of adults identify several risk factors that are more unique to adult victim populations, including needing to financially support dependents, low educational attainment, and having few job skills (Holger-Ambrose et al., 2013). Despite differences in adult and underage victim populations, much of the extant research on recruitment for sex trafficking have pooled both underage and adult participants or examined victimized adults only, limiting our understanding of the STM specifically (Reid, 2014). In order to translate sex trafficking research into evidence-based initiatives to combat the STM, it is important for research to delineate the specific ways in which traffickers target and recruit youth into the sex trade. The current study aims to synthesize research that focuses on youth recruitment into sex trafficking in North America.

## North American Context of Sex Trafficking

There have been few attempts to estimate the prevalence of the STM in North America; however, available statistics are often “guesstimates” rather than reliable rates (Franchino-Olsen et al., 2020; Stransky & Finkelhor, 2012). Available estimates for STM most commonly come from the United States, where the rates range from 1,400 to upward of 199,000 victims (Banks & Kyckelhahn, 2011; Estes & Weiner, 2001; Snyder & Sickmund, 2006; U.S. Department of Justice, 2004), with the most commonly cited study estimating upward of 325, 000 children at risk for sexual exploitation in the United States each year (Estes & Weiner, 2001). However, available statistics are problematic as they often fail to distinguish between domestic and international victims, are based on varying definitions of sex trafficking, are geographically limited, and utilize nonreplicable, unreliable methodologies (Fedina et al., 2019; Franchino-Olsen et al., 2020; Stransky & Finkelhor, 2012). Researcher error aside, the very nature of the sex trafficking industry presents barriers to the acquisition of accurate statistics. Most significant among these is the fact that trafficking occurs largely underground, within criminal networks that are transient, discrete, and often invisible, even to law enforcement (Duger, 2015; Franchino-Olsen et al., 2020). Difficulty in obtaining estimates of an invisible crime is compounded by the fact that many individuals victimized by sex trafficking do not view themselves as victims of a crime and therefore do not report it in any official capacity (Mcclain & Garrity, 2011). Despite flawed and unreliable statistics, STM is known to be widespread across Canada and the United States, requiring immediate action and sound research to uncover trends and pathways of youth into sex trafficking including the way traffickers target, recruit, and enmesh youth in the sex trade (Clawson et al., 2009; Cole & Sprang, 2015; Dalley, 2010).

While STM defies geographic borders, a country’s economic environment, geographic positioning, laws, employment rates, per capita income, and historical events shape the industry and individual risk for recruitment (Hepburn & Simon, 2010; C. O’Brien, 2009). As a result, trends in STM within North America are different from the European context. The permeable borders between European countries allow for easy international movement between proximal countries (Lindstrom, 2004). For example, one report found only 5% of all identified sex-trafficked victims in the United Kingdom (UK) were originally from the UK, which is a stark contrast to the picture of trafficking in NA where the majority of victims are domestic persons (Baird, McDonald & Connolly, 2020; Banks & Kyckelhahn, 2011; Mitchell et al., 2010; Royal Canadian Mounted Police [RCMP], Human Trafficking National Coordination Centre, 2014; Serious Organised Crime Agency, 2013). Given sex trafficking industries vary between countries based on differences in social, geographical, cultural, economic, and historical factors, it is not appropriate to generalize understandings of STM across countries that are dissimilar across these factors (Hepburn & Simon, 2010). As such, the current study narrowed its focus to systematically reviewing the recruitment of minors for sex trafficking in two countries, Canada and the United States both of which have similar cultural, economic, geographic, and historical contexts.

The domestic STM is of major concern within Canada and the United States (Clawson et al., 2009; Dalley, 2010). While both countries adhere to the standards of affluent and profitable nations that are alluring destinations for international sex traffickers, research consistently shows that domestic youth (i.e., youth trafficked within their country of origin) comprise the majority of underage victims in their respective countries (Baird et al., 2020; Kotrla, 2010; RCMP, Human Trafficking National Coordination Centre, 2014). Due to the risks and challenges associated with transporting victims across borders, some research suggests that domestic youths are preferred by traffickers (Smith et al., 2009). In summarizing the literature on recruitment and entrapment, it is important to distinguish between international and domestic sex trafficking due to the nuanced differences in the process of exploitation. Comparatively, researchers suggest domestic sex traffickers more often utilize interpersonal relationships and domestic violence to entrap their target and international traffickers rely upon kidnapping, parents’ selling their children, and offering false promises of jobs abroad for entrapment (Cecchet & Thoburn, 2014). Understanding the specific ways American and Canadian youths are recruited by traffickers and exploited domestically is important in developing effective prevention and intervention strategies.

## Current Study Goals

Given emergent issues of domestic STM in North America, federal and local governments wish to develop evidence-based approaches to combat sex trafficking and protect domestic youth. For example, a provincial government in Canada invested 307 million dollars toward antihuman trafficking initiatives, and the American federal government awarded over 100 million dollars for human trafficking initiatives and survivor supports. Research on STM has increased over the last decade, including the specific ways in which traffickers target, recruit, and exploit *youth* for commercial sexual exploitation. Recent research has also shifted to focusing on domestic trafficking rather than international trafficking from other countries, expanding our understanding of STM in North America. Understanding the process of recruitment for youth into sex trafficking is invaluable information for service providers and lawmakers developing prevention and intervention initiatives. The goal of this study was to systematically synthesize the research on the recruitment process employed by traffickers in the sex trafficking of domestic minors in the United States and Canada. Based on this synthesis, an overarching framework that explains the broader process of recruitment of domestic minors into sex trafficking is proposed.

## Method

This systematic review included articles that examine the recruitment process of domestic youth into sex trafficking within the American and Canadian context. Inclusion criteria were predetermined and documented in a protocol guiding the review process. The protocol was developed and agreed upon by both authors. To be eligible for inclusion in the present review, studies were required to be original research studies with quantitative and/or qualitative data and analysis, published in English between January 1990 and June 1, 2020. Articles published from 1990 onward were selected as these articles would be reflective of the changes that ensued with the international agreement and signing of the United Nations Convention on the Rights of the Child on the definition of the sexual exploitation of children. The selected period also represents the time frame when the majority of research on sex trafficking were published. Based on the geographical, social, and political similarities that impact criminal patterns of sex trafficking, the current study included studies focusing on minors trafficked within the United States and Canada. Studies were required to include data pertaining specifically to the recruitment and grooming of minors (youth under the age of 18), as previous research has highlighted that the recruitment of adults is different from minors (Bouché & Shady, 2017). Studies were excluded when the age-group of victims was unclear or when data were collapsed across ages. Given prior research suggests the trafficking of minors across borders (i.e., international sex trafficking) is qualitatively different from the domestic trafficking of minors (Cecchet & Thoburn, 2014), eligible studies for the current review specifically explored domestic sex trafficking, and articles were excluded if domestic and international trafficking cases were not parsed. A broad range of study designs and methodologies were included.

### Search Procedures and Study Selection

A comprehensive search was conducted in five electronic databases: PsycINFO, Sociological Abstracts, Social Services Abstracts, Applied Social Sciences Index and Abstracts, and Nursing and Allied Health Database. Alternate terms for sex trafficking were used in order to capture articles using delinquency language (e.g., teen prostitution) and consensual or transactional language (e.g., sex work, sex trade). Likewise, several terms were used to capture the concepts of trafficker recruitment and grooming strategies. Boolean operators “AND” and “OR” were used to combine sex trafficking, recruitment, and age terms. The search strategy comprised of the following key words: (“Human Traffick*” OR “Sex Traffick*” OR “Trafficking in Persons” OR “commercial sex* exploit*” OR child sex* exploit* OR “anti-trafficking” OR Prostitut* OR “Sex Work” OR “Domestic minor sex trafficking” OR “DMST” OR “sex trade” OR “sex* exploit*”) AND (coerc* OR “coercive control*” OR recruit* OR groom* OR entrap* OR deceit OR deception OR forc* OR threat* OR “sexual grooming” OR “abuse of power”) AND (“young person” OR juvenile OR “young people” OR minor* OR child* OR teen* OR adolescen* OR youth* OR “under 18” OR “at risk populations” OR “predelinquent youth”). Additional publications and gray literature were identified through a backward search of the references cited in articles reviewed for inclusion.

The initial screening of titles and abstracts was completed by the first author. Articles were excluded during this phase if obviously ineligible. Full-text screening was completed by two reviewers independently. Both reviewers screened the full text of articles retrieved to assess eligibility. Reviewer decisions were compared, and discrepancies were resolved through discussion. A third reviewer was included when consensus was not reached.

## Results

### Search Results

**Figure 1.**
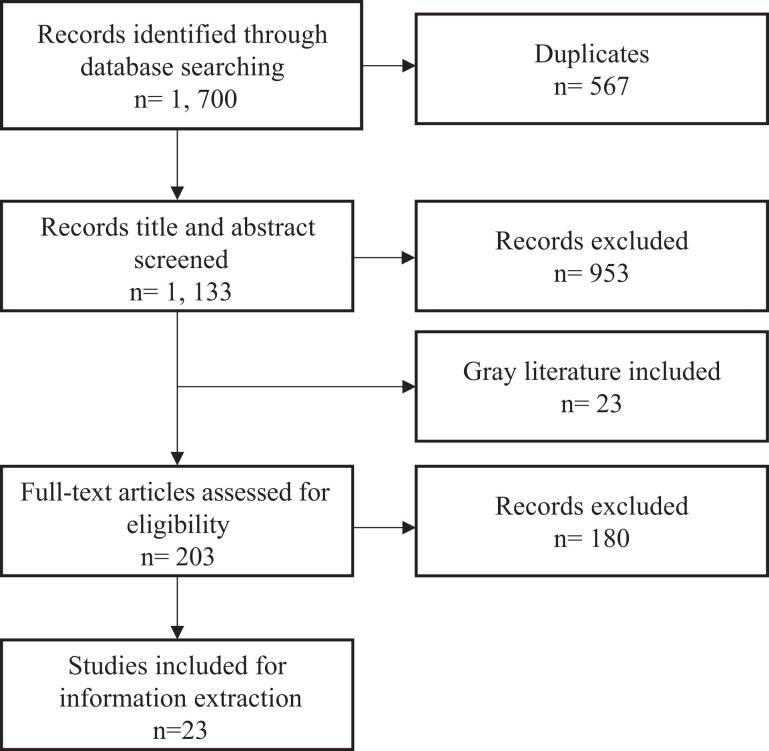
Preferred Reporting Items for Systematic Reviews and Meta-Analyses flow diagram.

Database searches generated 1,700 publications, of which, 1,133 were unique publications after removing duplicates (*n* = 567). The initial title and abstract screen eliminated 953 articles; 180 articles were retrieved for full-text screening, and an additional 23 articles from the gray literature were included for the full-text screen. After the two reviewers independently reviewed the remaining 203 articles, 180 were excluded due to not meeting one or more of the following eligibility criteria: did not speak about sex trafficking (e.g., exclusive focus on rape or labor trafficking) or recruitment and entrapment strategies (*n* = 117), adult and minor victim samples were grouped in the study (*n* = 9), study grouped international and domestic trafficking samples (*n* = 21), the study samples were not Canadian or American (*n* = 8), or were duplicate reports (*n* = 2). Book and movie reviews, opinion pieces, systematic and scoping reviews, and all other study designs that did not involve the analysis of primary or secondary data were excluded (*n* = 23). While many studies were excluded for failing to meet several eligibility criteria (e.g., grouping adults and minors and location of study), if the article did not study sex trafficking or recruitment and entrapment strategies, it was excluded primarily on these criteria alone. In total, 23 studies met our inclusion criteria (see Figure 1).

### Study and Sample Characteristics

Eighteen peer-reviewed articles, two government reports, and three dissertations met inclusion criteria. Twenty of twenty-three studies utilized American samples, and three used Canadian samples. Thirteen studies used qualitative methods, nine of which involved retrospective interviewing. Seven articles involved mixed method designs, and three utilized secondary data analysis. See Table 1 for a summary of study characteristics across studies. The majority of included studies were published in 2012 or after (*n* = 19), and the remaining studies were published in 2002 (*n* = 1), 2009 (*n* = 2), and 2010 (*n* = 1). The 23 studies included data from individuals trafficked as minors (*n* = 18), stakeholders (*n* = 7), and active or previously self-identified pimps (*n* = 2), with some studies using data from two of the aforementioned three categories. Stakeholders included government agency, law enforcement, nongovernmental organization (NGO) representatives, and adolescents with knowledge about the domestic STM (i.e., having participated in selling sex, provided services to sexually exploited youth, or knew a family member involved).

**Table 1.** The Exploitation Continuum: Summary of Critical Findings.

The Recruitment Context
Who are traffickers? – Traffickers are most commonly male. – Traffickers and recruiters are most commonly cited as romantic partners or friends. – Other cited relationships including family, roommates, schoolmates, boyfriend of a friend, buyers or “Johns,” employers, drug dealers, and strangers. Youth characteristics – Youth with unmet financial, love, and belonging or basic needs. – Child welfare involvement and experiences of childhood maltreatment. – Runaway youth and youth in homeless shelters. – Other cited youth risk factors include drug addiction, independent sex work, being First Nations or Indigenous, and having an intellectual disability. Initial location of recruitment – Most commonly cited recruitment location is online. – Other locations include bus stops, homeless shelters, outside schools, malls, nightlife, social gatherings, employment, in the neighborhood, at the park, at corner stores, or even within their own home.
Methods of entrapment
Relational tactics – Boyfriend scheme (i.e., “Romeo pimping”) most commonly cited tactic. ˆ Boyfriend recruiters/traffickers “sell the dream” of a life together, provide unmet needs, and groom with attention, love, gifts, drugs, money. ˆ Eventual shift from romance to exploitation involves manipulation and/or force. – “Befriending” tactic may include other girls working for trafficker pose as friend in recruitment, or friends may normalize selling sex, operate as a role model in sex trade, or use peer pressure to get youth to sell sex. – “Familial pimping” is cited as the most coercive and accounts for the youngest victims, where parental authority and family loyalty act as coercive strongholds. Aversive tactics – Aversive tactics include blackmail, financial abuse, pushing sexual boundaries, abduction, torture, drugging, gang rape, removing youths’ identification, threats, and sexual violence in forcing youth to have sex with men for money. – Aversive tactics used during shift from grooming to violence or upon first encounter.
Enmeshment process
– Control tactics: Fear, shame, feeling “owned,” experiencing threats, intimation, blackmail, systemic isolation, trauma bond, loyalty, to trafficker. – Dependency factors: Trafficker nurturing drug addiction, and trafficker is sole provider of basic needs, pregnancy, and debt bondage. – Youth factors: Youth relationship/attachment to trafficker, need for love, and increased agency in sex work.

Eighteen of the 23 studies include demographic information on victims or survivors of sex trafficking. Overall, studies included African American or Black (*n* = 13 studies), Caucasian or White (*n* = 13), Hispanic (*n* = 7), Asian American (*n* = 2), South Asian (*n* = 1), Indigenous or Native (*n* = 3), mixed race (*n* = 8), and other (n = 1). Eleven articles reported on the gender of victims. Samples were predominantly all female (seven of 11 studies), with only four studies including male or transgendered participants. Only two articles provided data on sexual orientation.

## Findings

Findings from the reviewed articles support an exploitation continuum comprising of three components: the recruitment context, methods of entrapment, and enmeshment.

### The Recruitment Context

#### Characteristics of traffickers

In the articles reviewed for this article, traffickers, pimps, and recruiters are terms used interchangeably to define the individual(s) who initially introduce the minor into the sex trade. Traffickers or pimps are described to either take on the recruiting role themselves or use recruiters to seek out vulnerable youth (Dalley, 2010; Williamson & Prior, 2009). Williamson and Prior (2009) note traffickers may use previously exploited youth to recruit. Traffickers were identified as most commonly male; however, female traffickers were also reported and participate in the recruitment and exploitation of minors (Reid, 2016; Roe-Sepowitz, 2019; Williamson & Prior, 2009). Females may also be involved in the exploitation of youth as recruiters who work for a trafficker. Females in this role, commonly referred to as the “bottom girl,” are often exploited themselves by the trafficker and attain relative status within the trafficker’s network by taking on responsibilities of recruiting new girls into their “stable” (Roe-Sepowitz, 2019; Smith et al., 2009; Williamson & Prior, 2009). Four articles highlight that traffickers may also be gang member or organized crime affiliates (Dalley, 2010; Reid, 2016; Roe-Sepowitz, 2019; Smith et al., 2009).

#### Youth relationship with trafficker

Several different types of relationships between the trafficker/recruiter and youth are cited by the reviewed articles, the most commonly cited relationship being a romantic one as cited among 18 of the 24 reviewed studies (Anderson et al., 2014; Bruhns et al., 2018; Baird et al., 2020; Cecchet & Thorburn, 2014; Corbett, 2018; Dalley, 2010; Edinburgh et al., 2015; Gibbs et al., 2015; Moore et al., 2020; Nixon et al., 2002; Perkins & Ruiz, 2017; Reed et al., 2019; Reid, 2016; Rosenblatt, 2014; Smith et al., 2009; Tidball et al., 2016; Wells et al., 2012; Williamson & Prior, 2009). Three studies identify “boyfriends” as the most common recruiter of youth into the sex trade (Gibbs et al., 2015; Rosenblatt, 2014; Smith et al., 2009), particularly among older youth aged 16–17 (Moore et al., 2020). The second most commonly cited relationship type is a friend (15 studies; Baird et al., 2020; Bruhns et al., 2018; Cavazos, 2015; Cecchet & Thoburn, 2014; Corbett, 2018; Dalley, 2010; Marcus et al., 2014; Moore et al., 2020; Nixon et al., 2002; Perkins & Ruiz, 2017; Reed et al., 2019; Reid, 2016; Rosenblatt, 2014; Smith et al., 2009; Wells et al., 2012), with two studies finding friends to be the most common recruiter (Moore et al., 2020; Nixon et al., 2002). Friends are identified as both male and female, long-time friends, or “false friends” met shortly before entrapment (Anderson et al., 2014; Corbett, 2018; Reed et al., 2019; Rosenblatt, 2014). Eleven studies cite family members to be recruiters or traffickers for the sexual exploitation of minors, including fathers, mothers, siblings, or foster parents (Baird et al., 2020; Bruhns et al., 2018; Cecchet & Thoburn, 2014; Corbett, 2018; Dalley, 2010; Edinburgh et al., 2015; Marcus et al., 2014; Perkins & Ruiz, 2017; Reed et al., 2019; Reid, 2016; Smith et al., 2009; Wells et al., 2012). Perkins and Ruiz (2017) distinguish between urban and rural youths, noting that rural youths tend to be trafficked by family members whereas urban youths tend to be trafficked by friends. Reid (2016) found mothers to be the most common type of family member initiating youth into the sex trade. Other cited relationships include roommates, schoolmates (Dalley, 2010), boyfriend of a friend (Perkins & Ruiz, 2017), buyers or “Johns,” employers, drug dealers (Marcus et al., 2014; Reid, 2016), and acquaintances (Moore et al., 2020; Nixon et al., 2002; Williamson & Prior, 2009). Five studies cite strangers, who have no prior bond with the youth, as recruiters (Baird et al., 2020; Moore et al., 2020; Reid, 2016; Rosenblatt, 2014; Wells et al., 2012). Rosenblatt (2014) highlighted a common tactic is for traffickers who are strangers to pose as a neighborhood boy, friend, or safe person in a local area.

#### Youth characteristics

Six studies reference the significance of sex-trafficked youth coming from impoverished households with unmet financial needs (Bruhns et al., 2018; Corbett, 2018; Moore et al., 2020; Reed et al., 2019; Rosenblatt, 2014; Smith et al., 2009). In addition, involvement with child welfare and childhood experiences of physical, sexual, and emotional abuse is common across many of the reviewed articles (Baird et al., 2020; J. E. O’Brien, 2018; Perkins & Ruiz, 2017; Reed et al., 2019; Rosenblatt, 2014; Smith et al., 2009; Tidball et al., 2016; Williamson & Prior, 2009). Seven studies emphasize that traffickers will identify and attend to youths’ unmet needs for love, care, and attention; low self-esteem; their desire to escape homelife; or need for a parent-like figure to fill their basic needs of food, clothing, security, and shelter (Anderson et al., 2014; Cecchet & Thoburn, 2014; Corbett, 2018; Moore et al., 2020; Perkins & Ruiz, 2017; Rosenblatt, 2014; Smith et al., 2009). Four studies identify that youth living in group homes and shelters are at enhanced risk for recruitment (Baird et al., 2020; Smith et al., 2009; Tidball et al., 2016; Williamson & Prior, 2009). Four studies pinpoint runaway youth or homeless youth as vulnerable to sex trafficking recruitment (Anderson et al., 2014; Perkins & Ruiz, 2017; Reed et al., 2019; Williamson & Prior, 2009). Similarly, Dalley (2010) highlights that youths leaving First Nations communities to larger cities are prime targets for recruiters and traffickers in Canada. In addition, Cecchet and Thoburn (2014) found sex-trafficked youth retrospectively reported having lived in neighborhoods with prostitution prior to being recruited themselves. Four studies classify risky youth behaviors such as substance use or addiction issues and independent sex work placing youth at risk for recruitment (Bruhns et al., 2018; Moore et al., 2020; Perkins & Ruiz, 2017; Reed et al., 2019). Youth with intellectual disabilities is cited as a group at enhanced risk for deception, manipulation, and exploitation by a trafficker (Dalley, 2010; Marcus et al., 2014; Reid, 2016). For example, Reid (2016) described a victimized youth with an intellectual disability struggling to decipher between a John and a boyfriend.

#### Location of initial recruitment

Six studies cite youth being recruited at various locations such as at bus stops, homeless shelters, outside juvenile justice centers, outside schools, malls, nightlife, social gatherings, employment, in the neighborhood, at the park, on the street, at corner stores, or even within their own home (Baird et al., 2020; Dalley, 2010; Moore et al., 2020; Rosenblatt, 2014; Smith et al., 2009; Williamson & Prior, 2009). However, initial contact via the internet was cited by more studies (*n* = 7) than other recruitment locations (Baird et al., 2020; Moore et al., 2020; J. E. O’Brien, 2018; J. E. O’Brien & Li, 2020; Rosenblatt, 2014; Tidball et al., 2016; Wells et al., 2012). Internet-facilitated recruitment occurs when traffickers access youth by frequenting online platforms popular with youth and using strategies such as initiating interpersonal relationships or even deceptively posing as an old friend (J. E. O’Brien & Li, 2020; Rosenblatt, 2014). Wells and colleagues (2012) noted online recruitment cases are more common among younger juveniles aged 14 and 15. Several specific internet-based applications are cited as locations where youth is recruited including Facebook, Snapchat, Tinder, Kik, Instagram, Whisper, Craigslist, and online multiplayer video games (Baird et al., 2020; J. E. O’Brien & Li, 2020; Tidball et al., 2016). Youth who posts sexually explicit images or independently sell sex online is targeted within these cyberspaces by pimps/traffickers (Dalley, 2010; J. E. O’Brien & Li, 2020).

### Methods of Entrapment in the Exploitation Continuum

Entrapment refers to the strategies or tactics used by traffickers to engage with youth and recruit them into sex trafficking (Baird et al., 2020; Reid, 2016). Two strategies emerged from the reviewed studies on how youth is entrapped by traffickers into sex trafficking: relational tactics and aversive tactics.

#### Relational tactics

Most commonly cited (15 studies) among all trafficker entrapment methods is the “boyfriend” scheme, also referred to as “romancing” or “Romeo pimping” (Anderson et al., 2014; Cavazos, 2015; Corbett, 2018; Dalley, 2010; Gibbs et al., 2015; Marcus et al., 2014; Moore et al., 2020; Reed et al., 2019; Reid, 2016; Roe-Sepowitz, 2019; Rosenblatt, 2014; Smith et al., 2009; Tidball et al., 2016; Wells et al., 2012; Williamson & Prior, 2009). Several articles suggest that traffickers target youths’ desire for love and belonging, particularly those who have unmet needs in this domain, by establishing a seemingly loving and caring relationship, under the guise of being a boyfriend (Cavazos, 2015; Rosenblatt, 2014; Smith et al., 2009). Smith and colleagues (2009) further elaborate that the romance scheme cultivates control over the victim by establishing an unwavering allegiance to the trafficker, making them more likely to succumb to propositions to sell sex. Furthermore, many of the articles suggest traffickers who pose as a loving boyfriend may “sell the dream” of a life together, appealing to the youths’ desires for stability, shelter, material things, and/or a lavish life (Anderson et al., 2014; Cavazos, 2015; Corbett, 2018; Dalley, 2010; Gibbs et al., 2015; Marcus et al., 2014; Roe-Sepowitz, 2019; Smith et al., 2009). Five articles make reference to some or all of the following: romancing or grooming the youth with attention, drugs, gifts, money, dates, intimacy, and asking her about her goals for the future (Baird et al., 2020; Corbett, 2018; Reid, 2016; Rosenblatt, 2014; Smith et al., 2009; Tidball et al., 2016). Gifts and money may function as a way to make the youth feel accomplished after the trafficker has sex with her and creating associations between sex and receiving material items or money (Smith et al., 2009). Several articles noted that the subsequent shift from romance to exploitation may involve force and/or coercion (Dalley, 2010; Edinburgh et al., 2015; Reed et al., 2019; Smith et al., 2009). Williamson and Prior (2009) coined the term “bait and switch” to describe this coercive tactic of appealing to youths’ unmet needs and subsequently exploiting them for their own financial gain. Other cited coercive strategies included propositioning the youth to selling sex as a way to make money for themselves (Marcus et al., 2014; Reid, 2016) or as a favor for the trafficker/boyfriend or their future together (Anderson et al., 2014; Gibbs et al., 2015). For example, a sex-trafficked youth may believe they are selling sex in order to raise money for the couple to buy a home and a car, believing in the possibility of marriage and kids at the end (Gibbs et al., 2015). Gibbs and colleagues (2015) describe a process whereby some youths are led to believe they are selling sex just one time for their boyfriend and are continually exploited thereafter.

Numerous articles (10 of 23 studies) reference “befriending” tactics utilized in the entrapment of youth into sex trafficking (Bruhns et al., 2018; Cavazos, 2015; Dalley, 2010; Edinburgh et al., 2015; Moore et al., 2020; J. E. O’Brien & Li, 2020; Reed et al., 2019; Reid, 2016; Rosenblatt, 2014; Smith et al., 2009). Two studies reference traffickers’ strategic use of other girls in his “stable” (i.e., children, youths, adults he is exploiting) to recruit new youth (Edinburgh et al., 2015; Smith et al., 2009). These girls are tasked with the job of befriending other girls, recruiting them for the pimp, training them, providing them with basic needs, and even advertising their services (Cavazos, 2015; Reed et al., 2019; Roe-Sepowitz, 2019; Smith et al., 2009). Peer recruitment is cited as particularly effective in the process of normalizing the selling of sex and operating as a role model by teaching youth the “ropes” of the sex trade (Moore et al., 2020; Reed et al., 2019; Reid, 2016; Roe-Sepowitz, 2019). For example, the female peer may suggest selling sex as a good way to make money and/or set up “double dates” to ease the new girl into independent sex work (Bruhns et al., 2018; Edinburgh et al., 2015; Reid, 2016). Alternatively, friends may use peer pressure as a way to get youth to conform and sell sex (Dalley, 2010; Edinburgh et al., 2015; Moore et al., 2020; Reed et al., 2019; Rosenblatt, 2014). Reid (2016) finds peers may appeal to prior drug addictions and suggest selling sex as a means to access drugs. Alternatively, J. E. O’Brien and Li (2020) describe that traffickers themselves may assume the peer/friend role during recruitment, online friendships, and arranging for in-person meetings are a common first introduction.

Less commonly referenced are biological family or foster-parent traffickers (five of 24 studies) who utilize their position of authority in entrapping the youth in sex trafficking (Corbett, 2018; Dalley, 2010). Two studies found that familial pimping accounted for the youngest victims (Marcus et al., 2014; Reid, 2016). Familial traffickers were cited to use force or coercion in the context of recruitment (Corbett, 2018; Dalley, 2010). According to Marcus and colleagues (2014), “familial pimping” is the most coercive type of relationship, with parental authority and family loyalty acting as coercive strongholds over the child (Marcus et al., 2014; Rosenblatt, 2014). In one case presented by Edinburgh and colleagues (2015), a mother initiated her daughter into the sex trade by coaching her on how to perform sexual services. Another case example by Marcus and colleagues (2014) recounted a foster father who initiated his foster daughter into commercial sex by insisting her to contribute to the household by having sex for money with his friends. Alternatively, nonfamilial traffickers may mimic parenting-like dynamics and a pseudo-family environment during recruitment (Rosenblatt, 2014; Smith et al., 2009).

#### Aversive tactics

According to Baird and colleagues (2020), the use of aversive tactics in the process of entrapment is less common than grooming and luring tactics such as providing the youth with gifts, drugs, and attention. Thirteen studies made reference to traffickers who utilize aversive tactics including violence and coercion (Baird et al., 2020; Bruhns et al., 2018; Cavazos, 2015; Dalley, 2010; Gibbs et al., 2015; Moore et al., 2020; Nixon et al., 2002; J. E. O’Brien, 2018; Reed et al., 2019; Reid, 2016; Roe-Sepowitz, 2019; Rosenblatt, 2014; Williamson & Prior, 2009). While some studies highlight a shift from grooming to violence, particularly in the context of a romancing recruitment strategy (Dalley, 2010; Edinburgh et al., 2015; Reed et al., 2019; Smith et al., 2009), other recruitment tactics involve traffickers who are violent from the onset, most commonly referred to as “Gorilla/Guerilla pimps” (Dalley, 2010; Gibbs et al., 2015; Williamson & Prior, 2009). Cited Gorilla pimp tactics include abduction, torture, drugging, gang rape, removing youths’ identification, threats, and sexual violence in forcing youth to have sex with men for money (Bruhns et al., 2018; Dalley, 2010; Moore et al., 2020; Nixon et al., 2002; Reid, 2016; Rosenblatt, 2014; Williamson & Prior, 2009). Three studies describe traffickers’ use of financial abuse where a youth is forced to sell sex for money as a way to pay back her trafficker-owed money for drugs or other goods and services (Nixon et al., 2002; Reed et al., 2019; Reid, 2016). For example, Reed and colleagues (2019) describe an instance where a youth began to sell sex as a way to pay back the trafficker for the drugs he provided her. Dalley (2010) discusses traffickers’ use of blackmail to compel youth into adhering to their demands to begin selling sex. For example, the trafficker may stage a gang rape, photograph the event, and then threaten to expose the pictures to family and friends (Dalley, 2010). Two studies reference more insidious aversive tactics including slowly pushing sexual boundaries or desensitizing youth to nonconsensual sex by repeatedly sexually abusing them (J. E. O’Brien, 2018; Smith et al., 2009). Roe-Sepowitz (2019) reports 18.1% of the study’s reviewed cases involved a trafficker using sexual assault to condition youth to nonconsensual sex during the process of entrapment.

### Enmeshment in Sex Trafficking

Reid (2016) explored recruitment of minors into sex trafficking and identified that within the continuum of recruitment, there was a distinct enmeshment process serving to prolong exploitation beyond the recruitment phase due to specific barriers from exiting. Of the reviewed articles, 17 supported this concept of enmeshment as distinct from the initial recruitment when detailing the ways youth is recruited into sex trafficking and exploited over a period of time (Anderson et al., 2014; Bruhns et al., 2018; Cavazos, 2015; Cecchet & Thoburn, 2014; Corbett, 2018; Dalley, 2010; Edinburgh et al., 2015; Marcus et al., 2014; Nixon et al., 2002; J. E. O’Brien & Li, 2020; Perkins & Ruiz, 2017; Reid, 2016; Roe-Sepowitz, 2019; Rosenblatt, 2014; Smith et al., 2009; Williamson & Prior, 2009). Three clusters of factors that facilitate enmeshment emerged from the literature: control factors, dependency factors, and youth factors. The former two themes refer to the trafficker-related factors that promote prolonged exploitation of youth. “Youth factors” refers to youth-driven barriers to exiting.

#### Control factors

Fifteen of the reviewed articles make reference to control factors whereby youth feels unable to leave their exploitive situation due to feeling fear, shame, or like they are “owned” by their trafficker (Anderson et al., 2014; Bruhns et al., 2018; Cavazos, 2015; Cecchet & Thoburn, 2014; Corbett, 2018; Dalley, 2010; Edinburgh et al., 2015; Marcus et al., 2014; Nixon et al., 2002; J. E. O’Brien & Li, 2020; Reid, 2016; Roe-Sepowitz, 2019; Rosenblatt, 2014; Smith et al., 2009; Williamson & Prior, 2009). Marcus and colleagues (2014) cite Gorilla pimps who use aversive tactics that induce fear as the most difficult to leave. Several studies reference specific control methods including rape, burnings, violence, psychological abuse, intimidation, withholding documents, threats to the youth or their family’s life, impregnating the youth, and threatening their pregnancy or child (Anderson et al., 2014; Cecchet & Thoburn, 2014; Corbett, 2018; Dalley, 2010; Edinburgh et al., 2015; Nixon et al., 2002; J. E. O’Brien & Li, 2020; Reid, 2016; Roe-Sepowitz, 2019; Rosenblatt, 2014; Smith et al., 2009). More specifically, Reid (2016) describes several intimidation tactics including forcing youth to watch others getting raped or being beaten until miscarriage. Two studies reference blackmail tactics, such as exposing/threatening to expose explicit images or making them complicit in a crime, to instill shame and fear to control youth over a long period of time (Dalley, 2010; Reid, 2016). A commonly referenced control method is systematic isolation by the trafficker. Traffickers will disorient youth by moving them around from place to place, assuming control of their cell phones, limiting access to the internet, confining them to hotel rooms, withholding documents, and moving them far away from family and friends (Bruhns et al., 2018; Cavazos, 2015; Cecchet & Thoburn, 2014; Dalley, 2010; J. E. O’Brien & Li, 2020; Reid, 2016). Three studies reference the initiation of the “trauma bond” as a powerful control factor (Reid, 2016; Rosenblatt, 2014; Smith et al., 2009). According to Smith and colleagues (2009), the trauma bond is facilitated by the cycling of intimacy and violence to establish coercive control. According to these three studies, the trauma bond drives loyalty to the trafficker and provides hope amid violence that the loving behaviors will return (Reid, 2016; Rosenblatt, 2014; Smith et al., 2009). Reid (2016) describes the loyalty youths have for their trafficker fosters’ feelings of obligation and responsibility for the well-being of their trafficker. For example, Reid (2016) described a youth’s feelings of responsibility for the arrest of their trafficker and subsequent refusal to testify against him.

#### Dependency factors

Eight studies make reference to dependency factors that serve as barriers to youth exiting the sex trade and leaving their trafficker (Cavazos, 2015; Corbett, 2018; Dalley, 2010; Edinburgh et al., 2015; Marcus et al., 2014; Reid, 2016; Roe-Sepowitz, 2019; Smith et al., 2009). Dependency factors cited in the articles include impregnating the youth, being the sole provider of basic needs, nurturing drug addiction (“drug bondage”), or creating “debt bondage” by requiring hefty exiting fees to leave or pay back old debts to the trafficker (Dalley, 2010; Edinburgh et al., 2015; Reid, 2016; Smith et al., 2009). In fact, drug addiction is one of the most commonly cited barriers to exit (Cavazos, 2015; Dalley, 2010; Edinburgh et al., 2015; Marcus et al., 2014; Reid, 2016; Roe-Sepowitz, 2019). According to two studies, traffickers may also supply youth with drugs in order to create dependency on them (Corbett, 2018; Roe-Sepowitz, 2019). Baird and colleagues (2020) found that youth with prior drug use was more likely to be supplied with drugs during recruitment.

#### Youth factors

Five studies reference youth factors that prolong exploitation and act as barriers to exiting (Cecchet & Thoburn, 2014; Marcus et al., 2014; Perkins & Ruiz, 2017; Reid, 2016; Rosenblatt, 2014). Three studies describe the youth’s relationships to their trafficker and/or other exploited youth which may resemble romantic, friendship, or family attachments as significant factors prolonging the exploitation of the youth and keep them enmeshed (Cecchet & Thoburn, 2014; Marcus et al., 2014; Reid, 2016). For example, youth was cited to stay with their trafficker due to a reluctance to leave their friend who was also involved (Rosenblatt, 2014). Contrary to the typical narrative of sex trafficking victimization, Marcus and colleagues (2014) highlight that nearly all of their participants had increasing agency over their sex work over time which acted as a barrier to exiting the sex trade, as youth sold sex independently or recruited pimps for the facilitation of their services and for protection. Perkins and Ruiz (2017) emphasize that victimized youths’ strong need for love was a barrier to exiting, noting that many youth felt love and care in the trading of sex for money, despite understanding the exploitive nature of the sex trade.

## Discussion

The increased attention and research on sex trafficking has begun to uncover the widespread and horrific nature of the crime. Existing data on the trends of STM identify domestic youth as the primary victims of sex trafficking within Canada and the United States, with the average age of recruitment between 12 and 14 years of age (Jordan et al., 2013; RCMP, Human Trafficking National Coordination Centre, 2014; Smith et al., 2009). STM is widespread across Canada and the United States, occurring in all regions including micropolitan, metropolitan, and rural communities (Cole & Sprang, 2015). Federal and local governments are aware of the emergent sex trafficking issues and are funding initiatives to protect youth. A necessary component of these efforts is an empirical understanding of the specific ways in which domestic youth is recruited for exploitation in order to inform prevention and intervention initiatives. The purpose of the review was to synthesize empirical studies presenting information on the recruitment of domestic minors into sex trafficking within North America.

The synthesis of the articles for the present review supports a notion of exploitation occurring on a continuum involving trafficker entrapment and enmeshment tactics to recruit youth and prolong exploitation. More specifically, the proposed exploitation continuum comprises of three distinct, but not mutually exclusive, components: the recruitment context, entrapment strategies utilized by traffickers, and enmeshment tactics. The concept of a continuum incorporates the understanding that exploitation occurs in sequence from targeting victims to victim entrapment and enmeshment in the sex trade. However, an exploitation continuum is not synonymous with an exploitive template used by traffickers for each victim. Rather, the reviewed articles highlighted that pathways into sex trafficking are individualized and strategically catered to each victim according to their context, vulnerabilities, and characteristics. Thus, the context of the youth shapes the trafficker’s use of entrapment and enmeshment tactics, with some of the same tactics for entrapment and the initial exploitation of youth being used to enmesh youth and keep them within the trafficker’s control. Importantly, the exploitation framework proposed by the present review fills a significant gap in the literature by identifying the specific ways in which traffickers target youth and exploit their vulnerabilities in facilitating their entrapment into sex trafficking as well as prolonging their exploitation through enmeshment.

While much of the extant research on sex trafficking pool samples of adult and minor victims, the present review supports the view that traffickers target youth-specific characteristics and contexts in the process of exploitation, and thus, exploitation should be understood within the youth context separately. Youths are prime targets for traffickers, as they are more dependent on others for basic needs than adults, and they possess developmental vulnerabilities that are easily targeted in the process of exploitation (Bruhns et al., 2018; Cole & Anderson, 2013; Schwartz, 2015). Findings from the reviewed articles highlight that traffickers use luring and manipulative strategies to specifically target groups of youth living in the most precarious of situations, with a history of adverse childhood experiences, unmet needs, and exhibiting risky behaviors (e.g., Baird et al., 2020; Bruhns et al., 2018; Cecchet & Thoburn, 2014; Dalley, 2010; Perkins & Ruiz, 2017; Reed et al., 2019; Reid, 2016; Smith et al., 2009). Youth-specific contexts targeted by traffickers include youth living in foster homes, group homes, or are runaways. Moreover, traffickers target locations where youth spends unsupervised time such as malls, around schools, parks, and above all, online (Baird et al., 2020; Dalley, 2010; Moore et al., 2020; Rosenblatt, 2014; Smith et al., 2009; Williamson & Prior, 2009). While it is clear that the characteristics and contexts targeted by traffickers in the reviewed articles are youth specific, future research should systematically compare the recruitment of adults and youth to further delineate the differences.

With the continuum framework as a background, findings of this review present an evidence-based understanding of pathways into STM which can inform prevention initiatives to eliminate recruitment. Prevention efforts may be most effective online, as online avenues were the most commonly cited location where a youth initially meets their trafficker or is recruited (Baird et al., 2020; Moore et al., 2020; J. E. O’Brien, 2018; J. E. O’Brien & Li, 2020; Perkins & Ruiz, 2017; Rosenblatt, 2014; Tidball et al., 2016; Wells et al., 2012). Youth is known to spend copious amounts of time each day online, connecting with individuals they don’t know, and some engage in risky online behaviors such as sending sexually explicit images that set the stage for recruitment (J. E. O’Brien, 2018; J. E. O’Brien & Li, 2020). Indeed, it is increasingly common for youth to have friends exiting only in cyberspace (Ybarra & Mitchell, 2008), and traffickers take advantage of the anonymity of online connections in their efforts to target and entrap youth (J. E. O’Brien & Li, 2020). With the increasing presence of technology in youths’ lives, the evolving creation of new online applications to connect with others, and evidence of trafficker infiltration of these online avenues, prevention initiatives should involve information sharing with youth, families, and agencies about internet safety, red flags, parental monitoring, and privacy online (J. E. O’Brien & Li, 2020).

Youth education on exploitive, unhealthy relationships emerged as a key direction for prevention based on the relational entrapment strategies identified in the reviewed articles. Several red flags can be extracted from the exploitive continuum proposed that should be incorporated into the education of youth. In particular, romantic partners were the most commonly cited recruiters or traffickers (e.g., Gibbs et al., 2015; Moore et al., 2020; Rosenblatt, 2014; Smith et al., 2009). The shift from a caring romantic relationship to an exploitative relationship is filled with warning signs such as promises that never come to fruition, being told they owe money to their boyfriend (i.e., debt bondage), having their phone taken away, being isolated from friends and family, being blackmailed, desensitizing youth to nonconsensual sex through rape, and violence (e.g., Anderson et al., 2014; Dalley, 2010; Gibbs et al., 2015; Nixon et al., 2002; Reed et al., 2019; Roe-Sepowitz, 2019; Smith et al., 2009). In fact, the shift can be insidious, with the trafficker using grooming strategies such as providing attention, drugs, gifts, money, dates, intimacy, and subsequently utilize various manipulative or coercive strategies to compel the youth to sell sex (Baird et al., 2020; Reid, 2016; Smith et al., 2009). Education on the risks of sex trafficking should be widespread across North America, and youth should be aware of the ways in which traffickers target youth, exploit their romantic desires, and shift the relationship into exploitation. It is the belief of the authors that schools, agencies such as child welfare, and homeless shelters are important venues for such knowledge dissemination, as this is where youth from the most vulnerable circumstances are located.

In addition to identifying directions for effective prevention, the continuum framework, and enmeshment tactics more specifically, can be used to inform intervention initiatives to extract youth from the sex trade and prevent reentry. The concept of enmeshment, a term coined by Reid (2016), within the proposed exploitation continuum highlights the ways in which traffickers cunningly cultivate dependency and control over their victims to prolong their exploitation. Trafficker enmeshment tactics draw attention to the complexity of sex trafficking victimization including the trauma endured by victims and the psychological attachments keeping youth controlled by their trafficker. For example, becoming pregnant, being financially or drug reliant on their trafficker, having hefty debts to pay their trafficker (i.e., exiting fee) are the dependency factors keeping youth exploited (e.g., Corbett, 2018; Dalley, 2010; Edinburgh et al., 2015; Marcus et al., 2014; Reid, 2016; Roe-Sepowitz, 2019; Smith et al., 2009). Alternatively, control factors such as violence, intimidation, blackmail, and systemic isolation leave youth feeling unable to leave their trafficker due to the feeling of fear, shame, or like they are owned by their trafficker (e.g., Bruhns et al., 2018; Cavazos, 2015; Marcus et al., 2014; J. E. O’Brien & Li, 2020; Reid, 2016; Williamson & Prior, 2009). The cycling of intimacy and violence by some traffickers can facilitate the development of a trauma bond, driving victim loyalty toward the trafficker and acting as a barrier to exiting (Reid, 2016; Rosenblatt, 2014; Smith et al., 2009). As a result of these dependency and control factors, youth may perceive a lack of alternatives to their life with the trafficker, making it challenging to take steps in leaving their exploitive relationship/situation (Cavazos, 2015; Marcus et al., 2014; Reid, 2016).

Agencies including law enforcement who are tasked with the extraction of youth from the sex trade should be assessing for these dependency and control factors keeping youth victimized and subsequently identifying ways they can holistically support youth and meet their needs to break the dependency on their trafficker. For example, providing youth with financial stability, housing, childcare, and mental health support are a few of the potential supports to be in place in the process of extraction. A cornerstone for effective holistic intervention initiatives is interagency collaboration to ensure survivors receive coordinated services. For example, the Human Trafficking Prevention Intervention Strategy is a Toronto-based collaboration between 20 agencies (e.g., homeless shelters, child welfare agencies, police services) with goals to build partnerships between sectors, coordinate care, and include improve community capacity and responses to sex trafficking. However, effective survivor interventions do not only require capacity for service; rather, a deep understanding of the psychological complexity of sex trafficking enmeshment can allow service providers to approach youth with empathy in their efforts to keep them safe and prevent reentry. Indeed, the present review offers this understanding, highlighting the barriers to exiting in financial, emotional, and psychological domains.

## Limitations

This review was conducted systematically and rigorously and followed the Preferred Reporting Items for Systematic Reviews and Meta-Analyses guidelines for systematic reviews; however, there are limitations to be considered. First, while the authors of this review conducted an extensive, multistrategy, high-sensitivity search to attain all possible studies, it is possible that not all available studies were found and included in this review due to terms not used in our search or databases unexplored. Second, during the review process, the authors emailed the authors of studies where the sample was unclear (e.g., Were adult victims pooled with minors? Were internationally trafficked victims included in the sample?); however, if no response was attained, those articles were excluded from the present review. Third, the review only included studies published in the English language due to a lack of translation resources.

## Conclusions and Future Directions

This review presents the first synthesis of research on the recruitment process of domestic North American youth for sex trafficking. Importantly, the review fills a significant gap in the literature by identifying a framework for the exploitation continuum that involves a series of entrapment and enmeshment tactics that intersect with youths’ vulnerabilities and circumstances. Information derived from the studies draws attention to the vast and brutal experiences of victimized youth and the circumstances, making it challenging for youth to leave their trafficker. This study should serve as a call to action for governments, agencies, NGOs, and frontline workers to use empirical evidence to support their programs, policies, and practices. Illuminated by this study are the large hurdles faced by victimized youth to exit the sex trade and the multifaceted needs of survivors from the most basic, such as shelter, money, food, and clothing, to more complex psychological, medical, and emotional needs. Indeed, the unmet needs of survivors were the circumstances leaving them vulnerable to traffickers’ recruitment tactics in the first place. Therefore, policies and postexit programs need to support youth systematically and holistically in order to truly support their exiting.

An important backdrop to the issue of STM in North America is the shift in discourse from sex-trafficked youth as criminals (i.e., “teen prostitutes”) to survivors/victims of a crime which followed the criminalization of sex trafficking and federal consent laws between 2000 and 2008 in Canada and the United States. With a change in legislation came a shift in the way policy makers, law enforcement, and researchers began to view victimized youth as needing protection rather than punishment. While beyond the scope of the current article, it is interesting to note a clear acceleration of research on the recruitment and enmeshment of minors involved in sex trafficking following the legislative changes promoting the decriminalization of victims. In fact, all articles included in the present review were published 2002 and later, after the passing of the TVPA in 2000. Future research should further examine the political and practical implications of this shift in discourse.

While research on risk and vulnerability have highlighted the enhanced risk for LGBTQIA+ youth for recruitment into sex trafficking (e.g., Choi, 2015; Fedina et al., 2019), the reviewed articles largely failed to include diverse samples of youth identifying as LGBTQIA+. Thus, it remains unclear whether traffickers utilize similar or different tactics in the recruitment, entrapment, and enmeshment of LGBTQIA+ youth. Future research should focus on understanding the pathways to exploitation and experiences of LGBTQIA+ youth involved in sex trafficking. By delineating the unique experiences and needs of diverse youth populations exploited by sex trafficking, prevention efforts and intervention programs can better tailor their services to reflect the potentially unique needs of exploited youth. Future research on sex trafficking should also continue to explore the recruitment of domestic youth in North America and the role of familial trafficking, identify similarities and differences in the recruitment of adults and minors as well as domestic and international victims, and evaluate postexit programs aiming to support survivors.

## Implications for Practice, Policy, and Research

- Given the significance of online platforms for recruitment, prevention initiatives should take place online.
- Prevention initiatives should involve knowledge dissemination with youth, families, school staff, law enforcement, child welfare agencies, and homeless shelters about pathways into sex trafficking, internet safety, and red flags.
- Youth education on unhealthy and exploitive romantic relationships should be an important piece of academic curriculum in early adolescence.
- Interagency communication and collaboration is necessary to ensure survivors receive coordinated services.
- Policy attention is needed in efforts to provide victimized youth with financial stability, housing, childcare, and mental health support.
- Regional law enforcement agencies should develop specific human trafficking divisions that are well educated and equipped to support the extraction of youth from sex trafficking.
- Policy attention and funding are needed to support the development of trauma-informed aftercare for youth survivors of sex trafficking.
- We call on researchers to study the pathways to exploitation and experiences of LGBTQIA+ youth involved in sex trafficking.
- Future studies need to further delineate the route of exploitation by familial traffickers and identify similarities and differences in the recruitment of adults and minors as well as domestic and international victims.
